# Lack of Antilipoprotein Lipase Antibodies in Takayasu's Arteritis

**DOI:** 10.1155/2009/803409

**Published:** 2009-07-12

**Authors:** Jozélio Freire de Carvalho, Rosa Maria Rodrigues Pereira, Vilma Santos Trindade Viana, Eloísa Bonfá, Yehuda Shoenfeld

**Affiliations:** ^1^Disciplina de Reumatologia, Faculdade de Medicina da Universidade de São Paulo, 0124-6903 São Paulo, SP, Brazil; ^2^Department of Medicine B, Center for Autoimmune Diseases, Sheba Medical Center, 52621 Tel-Hashomer, Israel

## Abstract

*Background*. Antilipoprotein lipase (anti-LPL) antibodies were described in rheumatic diseases. In systemic lupus erythematosus they were highly associated with inflammatory markers and dyslipidemia, and may ultimately contribute to vascular damage. The relevance of this association in Takayasu's arteritis, which is characterized by major inflammatory process affecting vessels, has not been determined. *Objectives*. To analyze the presence of anti-LPL antibodies in patients with Takayasu's arteritis and its association with inflammatory markers and lipoprotein risk levels. *Methods*. Thirty sera from patients with Takayasu's arteritis, according to ACR criteria, were consecutively included. IgG anti-LPL was detected by a standard ELISA. Lipoprotein risk levels were evaluated according to NCEP/ATPIII. Inflammatory markers included ESR and CRP values. *Results*. Takayasu's arteritis patients had a mean age of 34 years old and all were females. Half of the patients presented high ESR and 60% elevated CRP. Lipoprotein NCEP risk levels were observed in approximately half of the patients: 53% for total cholesterol, 43% for triglycerides, 16% for HDL-c and 47% for LDL-c. In spite of the high frequency of dyslipidemia and inflammatory markers in these patients no anti-LPL were detected. *Conclusions*. The lack of anti-LPL antibodies in Takayasu's disease implies distinct mechanisms underlying dyslipidemia compared to systemic lupus erythematosus.

## 1. Introduction

Antilipoprotein lipase (anti-LPL) antibodies have been recently described in rheumatic diseases, mainly in systemic lupus erythematosus (SLE) and systemic sclerosis (SSc) but also in polymyositis and rheumatoid arthritis [1]. Lipoprotein lipase is a key enzyme in triglycerides metabolism and the presence of these autoantibodies might explain dyslipoproteinemias in these diseases. In fact, anti-LPL antibodies were identified in approximately 40% of lupus patients [1, 2] and were associated with high triglycerides (TGs) levels [2]. In addition, the influence of these antibodies on triglyceride metabolism was also confirmed in SSc since LPL activity was inhibited by anti-LPL in SSc sera of patients with elevated triglyceride levels [3].

 The presence of cellular infiltrates in vessels wall, as well as, the overexpression of cell-adhesion molecules on the endothelial cell membrane is a hallmark of chronic inflammatory disorders such as Takayasu's arteritis [4]. Since lipoprotein lipase is localized on endothelial surface of all vascular system carrying out triglycerides hydrolysis [5], it is therefore reasonable to speculate that the lipoprotein lipase may be involved in the inflammatory vascular process of Takayasu's arteritis [6, 7].

The present study was undertaken to determine the presence of antilipoprotein lipase antibodies and its possible link with dyslipoproteinemia and inflammatory markers in patients with Takayasu's arteritis.

## 2. Material and Methods

### 2.1. Patients

A total of thirty premenopausal female Takayasu's arteritis (TAs) patients that satisfied the proposed criteria of American College of Rheumatology for [8] were consecutively enrolled, from the Outpatient Clinics at the Rheumatology Division of São Paulo University Medical School. At entry, all patients were clinically evaluated, their medical records were extensively reviewed and a blood sample was collected. 

Rigorous exclusion criteria of conditions that interfere in the lipid profile, such as diabetes mellitus, pregnancy, menopause, liver or thyroid disease, ingestion of lipid-raising drugs or use of statins, were applied. This study was approved by the Local Ethical Committee.

### 2.2. Laboratory Evaluation

Serum samples were obtained from all patients after a 12-hour overnight fast after inclusion. Immunological and biochemical analysis were performed in the same serum samples. 

### 2.3. Assay for Antibody to Lipoprotein Lipase (LPL) Detection

Anti-LPL reactivity of IgG isotype was measured by Enzyme-linked immunosorbent assay (ELISA), as previously described [2]. Briefly, wells of Costar polystyrene plates were coated overnight with commercially available LPL from bovine milk (5 *μ*g/mL) (Sigma Chem. Co., St Louis, Mo, USA). Test was performed with serum samples 1/100 diluted in Tris buffered-saline containing adult bovine serum. Anti-LPL IgG isotype antibodies were determined with alkaline-phosphatase conjugated goat antihuman IgG (Sigma Chem. Co., St Louis, Mo, USA). The reaction was developed with p-nitrophenyl phosphate and optical density (OD) read at 405 nm with a Labsystems Multiskan MS (Helsinki, Finland). IgG anti-LPL positivity was defined for serum samples OD values ≥ 3SD above the mean OD of 20 healthy control serum samples included in each assay.

### 2.4. Lipid Profiles

Total cholesterol (TC) and triglycerides (TG) were measured enzymatically (Boehringer Mannheim, Argentina and Merck, Germany, resp.) on an RA 1000 analyser (Technicon Instruments Corp) [9, 10]. High-density lipoprotein cholesterol (HDL) was obtained after precipitation of very low-density lipoprotein cholesterol (VLDL) and low-density lipoprotein cholesterol (LDL) by phosphotungstic acid and magnesium chloride [11]. VLDL and LDL were estimated since all samples had triglycerides less than 400 mg/dL [12]. VLDL levels were calculated from the division of serum triglyceride by 5 (TG/5) [12] and LDL levels were estimated using the equation: LDL = TC − (HDL + TG/5) [12]. Risk lipoprotein levels were determined according to National Cholesterol Education Program-Adult Treatment Panel III (NCEP/ATPIII) [13].

### 2.5. Inflammatory Markers

C-reactive protein (CRP) serum levels were systematically determined in all patients by nephelometry. Erythrocyte sedimentation rate (ESR) was measured by modified Westergren method. These parameters were considered to be altered when CRP > 5 *μ*g/mL and ESR > 25 mm/first hour.

### 2.6. Statistical Analysis

Results are presented as the mean ± SD and percentage. Correlations among inflammatory markers and lipoprotein risk levels were calculated using Spearman's rank correlation test. *P*-values less than 0.05 were considered significant.

## 3. Results

### 3.1. Demographic Features

Takayasu's arteritis female patients (*n* = 30) had a mean age of 33.8 ± 8.1 years and 70% were white race. All patients had body mass index below 30 kg/m^2^ and the mean disease duration was 6.7 ± 6.9 years. More than half (57%) of patients studied were in use of prednisone but low daily doses were observed in TA patients (9.7 ± 13.7 mg/d). Systemic arterial hypertension (Blood pressure ≥ 140 × 90 mmHg, or antihypertensive use) was observed in eighty percent of TA patients (Table 1).

### 3.2. Lipoprotein Profile

In these premenopausal TA patients, the mean levels of total cholesterol were 205.7 ± 35.5 mg/dL, HDL-c 57.1 ± 18.7 mg/dL, and LDL-c 122.8 ± 31.2 mg/dL, and triglyceride was 126.7 ± 61.3 mg/dL. 

Moderate/high lipoprotein risk levels according to NCEP/ATPIII were observed in these subjects due to high total cholesterol in 53.3% (*n* = 16), low HDL-c in 16.6% (*n* = 5), high LDL-c in 46.6% (*n* = 14), and high triglyceride levels in 43.3% (*n* = 13). In addition, approximately half of the TA patients presented any lipoprotein risk levels (Table 2). 

### 3.3. Antilipoprotein Lipase Antibodies

No antilipoprotein lipase antibodies measured by ELISA was detected in TA patients.

### 3.4. Inflammatory Parameters

TA patients exhibited a more pronounced inflammatory alterations and had a mean ESR of 25.8 ± 17.6 mm/1st hour and a mean CRP of 11 ± 12.4 mcg/mL. Moreover, increased levels of CRP were observed in 60% of TA group and 50% presented elevated ESR (Table 3). 

A correlation analysis performed between inflammatory parameters and lipoproteins showed a significant negative correlation between CRP and HDL-c (*r* = −0.40; *P* = .028) but not with triglycerides (*r* = 0.16; *P* = .39), total cholesterol (*r* = −0.18; *P* = .34), and LDL-c (*r* = −0.007; *P* = .96). 

## 4. Discussion

Results from the current study showed that patients with Takayasu's arteritis do not present antibodies to lipoprotein lipase, although about seventy percent of them had at least one lipid risk levels for cardiovascular disease.

Importantly, we have selected only premenopausal female since the lipid profile is compromised due to the estrogen deficiency [14–16] and also gender is an important parameter that accounts for dyslipidemia studies [14, 16]. Importantly, all our patients had body mass index lower than 30 kg/m^2^, since obesity is a well known factor that modifies the lipid profile [14]. 

Moreover, the rigorous exclusion criteria used to select Takayasu's patients without other conditions that could interfere with lipid metabolism, such as, diabetes, thyroid disease, renal and hepatic involvements provided an opportunity to study the role of anti-LPL exclusively on Takayasu's disease without the influence of external factors [16–19]. 

Analysis of lipid profile revealed that in the patients with Takayasu's disease present lipid risk levels for cardiovascular disease due to high total cholesterol, high LDL-c and high triglycerides and low-HDL-c. These findings were similar to those found in chronic inflammatory disorders associated with atherosclerosis [20–22] such as systemic lupus erythematosus (SLE). 

Anti-LPL autoantibodies have been implicated in the inflammatory mechanisms of atherosclerosis in SLE and other inflammatory autoimmune disease [1, 3] with an evident vascular damage [23]. These antibodies were closely associated to the elevated levels of triglycerides in SLE and SSc and a putative functional role of anti-LPL in these diseases came from the observation that IgG fraction from SSc patient anti-LPL positive and with elevated serum triglyceride levels was able to significantly inhibit in vitro the enzyme activity [3]. Also, in our previous study that evaluated 66 SLE patients, with exclusion causes of dyslipidemia, we found positive anti-LPL in 37.8% of them. Moreover, this study showed a strong correlation between antilipoprotein lipase antibodies and CRP [2]. 

Although a high frequency of elevated inflammatory markers was observed in our TA patients, as well as a negative correlation between CRP with HDL-c, similar to found in lupus [2], the absence of a reactivity to lipoprotein lipase suggests that anti-LPL antibodies are not implicated in the ongoing active complex inflammatory process causing vascular damage in TA. 

In this aspect, it is reasonable to speculate that inflammatory and dyslipidemia processes occurring in TA and in systemic connective tissue diseases (SLE and SSc) may reflect diverse pathogenic mechanisms. In fact, systemic vasculitis are well known pauci-immune pathophysiological conditions. On the other hand, Shoenfeld et al. have previously demonstrated that antiendothelial cell antibodies may directly stimulate endothelial cells in TA through elevation of adhesion molecule expression associated with NF-kappaB activation and adhesion of monocytes, and may therefore play a pathogenic role in the development of the vasculopathy in this systemic disorder [24–26].

The present data are conclusive in showing that anti-LPL antibodies are not implicated in the pathophysiology of inflammatory atherosclerosis processes of Takayasu's arteritis.

## Figures and Tables

**Table 1:** Demographic, clinical features and prednisone use of patients with Takayasu's arteritis. Values are expressed in mean ± SD or percentage (%).

	Takayasu's arteritis (*n* = 30)
Age (years)	33.8 ± 8.1
White race, *n* (%)	21 (70)
Body mass index (kg/m^2^)	25.5 ± 5.5
Disease duration (years)	6.7 ± 6.9
Prednisone use, *n* (%)	17 (56.6)
Current prednisone dose (mg/day)	9.7 ± 13.7
Systemic arterial hypertension, *n* (%)	24 (80)

**Table 2:** Risk levels of Takayasu's arteritis patients according to NCEP/ATPIII. HDL-c = high-density lipoprotein cholesterol; LDL-c = low-density lipoprotein cholesterol.

	Takayasu's arteritis (*n* = 30)
Total cholesterol > 200 mg/dL, *n* (%)	16 (53.3)
HDL-c < 40 mg/dL, *n* (%)	5 (16.6)
LDL-c > 130 mg/dL, *n* (%)	14 (46.6)
Triglycerides > 150 mg/dL, *n* (%)	13 (43.3)
Any lipoprotein NCEP/ATPIII risk level, *n* (%)	20 (66.6)

**Table 3:** Inflammatory markers in patients Takayasu's arteritis. Values are expressed in mean ± SD or percentage (%).

	Takayasu's arteritis (*n* = 30)
C-reactive protein (mcg/mL)	11 ± 12.4
Erythrocyte sedimentation rate (mm/1st h)	25.8 ± 17.6
C-reactive protein >5 mcg/mL, *n* (%)	18 (60)
Erythrocyte sedimentation rate >25 mm/1st h, *n* (%)	15 (50)
